# Anthropometric failures and its associated factors among preschool-aged children in a rural community in southwest Ethiopia

**DOI:** 10.1371/journal.pone.0260368

**Published:** 2021-11-29

**Authors:** Kebebe Bidira, Dessalegn Tamiru, Tefera Belachew

**Affiliations:** Department of Nutrition and Dietetics, Institute of Health, Jimma University, Jimma, Ethiopia; Yenepoya Medical College, Yenepoya University, INDIA

## Abstract

**Background:**

In 2019, 144 million under-five-year-old children were stunted, and 47 million were wasted globally. In Ethiopia, approximately 350,000 children are estimated to die each year. Preschool aged children need focused attention because this age group not only has special needs, but also forms the platform for growth and development of all children. Under nutrition among preschool children is the result of a complex interplay of diverse elements, such as birth weight, household access to food, availability and use of drinking water. This study aimed at determining the anthropometric *failures* and associated factors using composite indictors.

**Methods:**

A community-based cross-sectional study design was used among randomly selected 588 caregivers with pre-school aged children. Under-nutrition of pre-school aged children was computed by using the composite index of anthropometric failure. A multi-stage sampling technique followed by a systematic random sampling technique was used to select study participants. Structured questionnaires were used to collect data. WHO Anthro software was used to calculate height for age, weight for age and weight for height. The overall prevalence of anthropometric failure (CIAF). Both bivariable and multivariable binary logistic regressions were used to identify factors associated with under-nutrition.

**Results:**

The overall prevalence of under-nutrition among pre-school children was 50.8%, which was significantly associated with being a female (AOR = 1.51, CI: 1.076, 2.12), being from a large family (AOR = 1.78, CI: 1.19, 2.663), having acute respiratory infection (AOR = 1.767, CI: 1.216, 2.566), lack of improved source of drinking water (AOR = 1.484 CI: 1.056, 2.085) and poor dietary diversity score (AOR = 1.5, CI: 1.066, 2.112).

**Conclusions:**

The study area has a high prevalence of CIAF in pre-school aged children. The CIAF was found to be significantly associated with the sex of the child, family size, ARI within the last two weeks, and dietary diversity score. To promote the use of family planning and the prevention of infectious diseases, health education is required. The government should adapt CIAF as a metric for assessing children’s nutritional status.

## Introduction

Under-nutrition is a matter of concern worldwide and one of the most important public health problems that inhibits cognitive and physical development as well as contributes to child morbidity and mortality [1]. In 2019, 144 million under-five-year-old children were stunted, 47 million were wasted, and 38 million were overweight globally [2]. In the case of preschool age, in 2012 the study estimated in developing countries 183 million are underweight, 226 million are stunted and 67 million are wasted [3]. According to a World Bank report, Asia and Africa are home to under-five child malnutrition, with Asia accounting for 56% of stunted children and Africa accounting for 37% of stunted children and 25% of wasted children. The report showed that the global trend in stunting, wasting and underweight was continuously decreasing, but at a slow pace [4]. In Ethiopia, 37% of under-five children are stunted, 7% are wasted, and 21% are underweight in 2019 [5]. In Ethiopia, approximately 350,000 children are estimated to die each year, mainly from preventable and treatable and infectious diseases complicated by malnutrition [6].

The World Health Organization (WHO) recommends 20% low prevalence, 20–29% medium prevalence, 30–39% high prevalence, and 40% very high prevalence as cut-off values for public health relevance of stunting. The cut-off values for wasting are 5% acceptable, 5–9% poor, 10–14% serious, and 15% critical, whereas the cut-off values for underweight are 10% low prevalence, 10–19% medium prevalence, and 20–29% high prevalence [7].

Preschool aged children need focused attention because this age group not only has special needs, but also forms the platform for growth and development of all children. Under nutrition among preschool children is the result of a complex interplay of diverse elements, such as birth weight, household access to food, availability and use of drinking water, sanitation, child and maternal care [8]. Under-nutrition of preschool aged children is conventionally assessed using anthropometric indices such as height for age, weight for height and weight for age. Low height for age (stunting) is an indicator of chronic malnutrition which is manifested by poor skeletal growth. Low weight for age (wasting) is an indicator of acute malnutrition with loss of lean as well as fat mass. Low weight for age (underweight) is indicative of both acute and chronic malnutrition [9, 10].

The Composite index of anthropometric failure (CIAF) was developed by Svedberg et al, 2000 and modified by Nandy et al in 2005 to estimate the burden of under-nutritional, because none of the three conventional indices are able to provide the overall prevalence of under-nutrition in the population. This is due to the overlapping of these indices; it means that the same child may show signs of having two or more anthropometric failures simultaneously [9, 11]. Inadequate dietary intake, illness/disease, household food insecurity, inadequate child care and feeding practices, unhealthy household and surrounding environments, and inaccessible and often inadequate health care are among the causes of undernutrition among children under the age of five, according to UNICEF’s conceptual framework [12].

Studies showed that poor socio economic conditions, food insecurity, poor feeding practice, food diversity, meals frequency, maternal under-nutrition, maternal education and infectious diseases were risk factors for malnutrition in preschool-age children [13–16]. The World Health Organization (WHO) reports that under nutrition in children < 5 years of age is often associated with inappropriate feeding practices [17]. The World Bank also reported that poor diets in terms of diversity, quality and quantity, together with illness, are the major contributors to under-nutrition of children [4]. The other study conducted in Malawi shows that low dietary quality and quantity and inappropriate feeding practices can cause under-nutrition and poor nutritional status in early childhood is associated with growth faltering [18].

The Ethiopian government’s productive safety net initiative is a comprehensive and unique solution for addressing chronic food poverty in a systematic manner [19]. The initiative targeted low-income and food-insecure families [20]. With such a high degree of child malnutrition, extensive efforts are being made to address these issues by developing various programs such as the National Nutrition Strategy [21]. Children’s under-nutrition is still a big issue in Ethiopia. Numerous studies have been conducted among children under the age of five to assess the prevalence and factors associated with under-nutrition, but few studies have been conducted among preschool-aged children. However, instead of using CIAF, all of these studies in the country used traditional indices.

## Materials and methods

### Study design and setting

From March to April 2019, a community-based cross-sectional study was conducted among pre-school aged children in rural areas of Ilu Abba Bor Zone, Southwest Ethiopia. The Ilu Abba Bor Zone is 600 kilometers away from the country’s capital city. The Zone is divided into 14 districts, 13 of which are rural and one administrative town. The zone’s estimated total population was 934,783, with 153,585 children under the age of five and 100,209 children aged two to five years. It is located at 70 27’40 "N to 90 02’ 10" N latitude and 340 52’12 "E to 410 34’55" longitude in the western part of the area. There are three major climatic zones in the zone: temperate rainy, rainy, and dry arid climates. Rainfall occurs twice a year in the zone. The highest annual rainfall total reaches 2400 mm, while the lowest annual rainfall total is about 100 mm. In most highland areas of Ilu Abba Bora, the highest mean annual temperature ranges from 26°C to 10.6°C.

### Sample size determination and sampling technique

The sample size was calculated using a G-Power model with the following assumptions: 52.5 percent estimated prevalence of stunting among children aged 24 to 59 months, 2.07 odds ratio of acute respiratory tract infection [22], 80 percent power, and a 5% margin of error. As a result, the sample size is calculated to be n = 256. Then, when calculating the final sample size, the design effect of 2 and 15% non-response rate were taken into account. As a result, the total sample size was 588.

To find caregivers with preschool-aged children, researchers used a multi-stage sampling technique followed by a systematic random sampling technique. Four districts were selected at random from a total of fourteen districts in the first round. In the second level, kebeles (Ethiopia’s smallest administrative units) were chosen from the districts that had been chosen. Simple random sampling was used to choose twenty-two of the kebeles. Pre-school aged children (2–5 years) were classified and registered in each kebele after the kebeles were chosen. Following the registration of preschool-aged children, proportional allocation was used to pick a sample from each kebele using a systematic random sampling technique. If a caregiver/household had more than one child, a child was chosen at random via a lottery system. A second visit was made if the mothers/caregivers were not present at the time of data collection. If that didn’t work, the residents of the neighborhood were considered.

### Data collection instruments and procedure

Data were collected using a structured interviewer questionnaire. It was adapted from Ethiopia demographic health survey 2016 [23]. To maintain consistency, the questionnaire was first written in English and then translated into the local language (Afan Oromo). It was then translated back into English by an expert translator. Before the actual data collection, a pre-test of the questionnaire was conducted on 5% of the total samples from outside the research area to assess the acceptability and applicability of the tools and procedures. As data collectors, 12 degree graduate nurses were recruited, and six degree graduate public health officers were in charge of supervision. Both data collectors and supervisors received rigorous training over three days.

To avoid within-examiner error, all anthropometric measurements were taken by investigators, qualified supervisors, and data collectors. Before performing the measurement, the weight scale was set to zero with no item on it and put on a level surface. The data collection was supervised by a principal investigator and trained supervisors. They supervised and checked each questionnaire for completeness, irregularities, inconsistencies, and unusual answers, making immediate corrections. During data entry, computer frequencies were used to search for missing variables, outliers, and other errors. The original questionnaire was revised to correct any errors discovered at this time.

The questionnaire includes questions about socio demographic and economic factors, water and hygiene habits, maternal and child health, and child feeding habits. Face-to-face interviews were conducted at the mothers’/caregivers’ homes by trained data collectors. The age of the child was calculated using the child’s date of birth and the date of the interview. Where the exact date of birth was not registered or known with certainty, the caregiver was asked to guess based on a local events calendar. By subtracting the date of birth from the date of data collection, the child’s age was calculated using a precise day.

Using repeated hours of dietary recall, the dietary intake of children was measured by asking caregivers (two week days and one weekend days). Every day of the week was described, including fasting and feasting days. The children’s caregivers were asked to remember all their child ate or drank during the 24-hour period of study. The Dietary Diversity Score (DDS) of the study respondents was calculated using the data from the 24-hour recall, according to the FAO guidelines for calculating household and individual dietary diversity [24].

Dietary diversity was calculated as the sum of scores in each of the seven food groups, with a scale of 0 to 7. The minimum dietary diversity (MDD) indicator was calculated using at least four of the seven food groups mentioned below: (1) staples (cereals/grains, roots and tubers); (2) dairy products; (3) animal/flesh foods (4) legumes and nuts; (5) vitamin A-rich fruits and vegetables; (6) eggs and (7) other fruits and vegetables [24]. The Ethiopia Demographic and Health Survey factors were used to create the Household Wealth Index, which is focused on household ownership of fixed assets, services, housing characteristics, and other factors [4].

### Anthropometric measurement

Standard anthropometric procedures were used to measure the children’s height and weight [25]. All randomly selected children who had no evidence of physical impairment (such as physical defects or grossly deformed), malnourished and edematous conditions were eligible for the weight and height measurements. The children’s heights were measured using a portable stadiometer.

All of the children were told to take off their shoes and stand erect, with their heels, knees, buttocks, shoulders, and heads in contact with the stadiometer’s wall and their eyes straight ahead (Frankfurt plane) so that their line of sight was perpendicular to their bodies.

To the nearest 0.1cm, the height was measured. A portable digital scale was used to measure the weight (Seca, Germany Model). To the nearest 0.1kg, the weight was registered. It was regularly calibrated against a known weight. During the training, a standardization exercise was conducted to capture technical measurement error before the actual anthropometric data collection (TEM). The children wore light clothing and removed their shoes for the procedure. Height for age Z-scores (HAZ), Weight for height Z-scores (WHZ), and Weight for age Z-scores (WAZ) were calculated using the height and weight measurements. In height for age, height for weight, and weight for age, moderately stunted, wasted, and underweight children had Z-scores of-3 to-2 SD. Stunted, wasting, and underweight children with Z-scores of less than-3SD are classified as severely stunted, wasting, and underweight [26].

The composite index of anthropometric failure was used to calculate the overall prevalence of under-nutrition in pre-school children (CIAF). The Nandy et al model was used to divide CIAF into seven (7) groups. Group A (no failure), group B (waste only), group C (waste and underweight), group D (stunting, wasting, and underweight), group E (stunting and underweight), group F stunting only), and group Y (underweight only) **(Table 1)** [27].

**Table 1:** Classification of composite index of anthropometric failure (CIAF)*.

Group	Description	Stunting	Wasting	Underweight
A	No failure	No	No	No
B	Wasting only	No	Yes	No
C	Wasting and underweight	No	Yes	Yes
D	Stunting, wasting and underweight	Yes	Yes	Yes
E	Stunting and underweight	Yes	No	Yes
F	Stunting only	Yes	No	No
Y	Underweight only	No	No	Yes

*adapted from Nandy et al, 2005.

### Data processing and analysis

Data was checked, cleaned, coded and entered into Epi-data 3.1 version and then it was exported to SPSS (version 21.0) for further analysis. Recoding and transforming of some variables were performed. Descriptive statistics such as frequencies and percentages for discrete data were calculated. Bivarable logistic regression was performed to identify potential candidate variables and each variable with a p-value less than 0.25 was interred into multivariable logistic regression analysis to determine the factors significantly associated with under-nutrition of preschool children.

Results with a P-value less than 0.05 were taken as statically significant and an Odds Ratio with 95% confidence interval (CI) was used to measure the level of association. Before the inclusion of predictors in the final model, standard error was used to assess for multicollinearity amongst predictor variables, and any variable with SE ≥0.2 was removed from the study. The goodness of fit for the final regression models was checked by the Hosmer-Lemeshow goodness of fit test with a p value of ≥0.05 considered a good fit.

For anthropometric data analysis, anthropometric indices were calculated by WHO Anthro software 3.2.2 using WHO child growth references. The z-scores of (< −2SD) were calculated to determine HAZ, WHZ and WAZ category of stunting, wasting and underweight, respectively. Finally, CIAF was computed from the above three anthropometric indices. Any child who has one of the six different types of anthropometric measurements is classified as having CIAF.

### Ethical considerations

Ethical approval was provided by the Jimma University Ethical Review Board. The letter was sent to the Ilu Abba Bora Zonal Health Department, as well as each selected district and kebele, in order to obtain consent. The mothers/caregivers of the children were given the requisite information about the study’s intent and procedures. Mothers/caregivers who agreed to participate in the intervention were requested to sign a written informal consent. All children who showed clinical symptoms of extreme malnutrition or a medical condition were referred to local health facilities for further evaluation and care.

## Result

### Socio-demographic characteristic of study participants

A total of 569 study subjects were involved in this study with a response rate of 96.1%. Among the mothers/caregivers who participated in the study, 263 (46.2%) were aged between 25–34 years. Of the total caregivers, 484 (85.1%) of them were married. Only 27 (4.7%) of the caregivers were educated till tertiary education. The majority of the caregivers, 386 (67.8%), were housewives, while 30 (5.3%) were government employees. Two hundred thirteen (37.4%) were Muslim religious followers. About 432 (75.9%) of the caregivers had a family size of <5 **(Table 2).**

**Table 2:** Socio-demographic characteristics of mothers/caregivers and their children in rural of Ilu Abba Bor Zone, Southwest Ethiopia, 2019.

Variables		Frequency(n = 569)	Percent (%)
Age of mother/ caregiver	<25	201	35.3
	25–34	263	46.2
	> = 35	105	18.5
Marital status	Single	40	7
	Married	484	85.1
	Widowed	17	3
	Divorced	28	4.9
	Divorced	28	4.7
Educational status of mothers/caregiver	Cannot read and write	91	16
	Can read and write	50	8.8
	Grade 1–4	91	16
	Grade 5–8	167	29.3
	Grade 9–12	143	25.1
	Tertiary education	27	4.7
	Famer	69	12.1
Occupation of the mothers/caregiver	Housewife	386	67.8
	Merchant	65	11.4
	Government employee	30	5.3
	Others*	19	3.3
Religion	Orthodox	137	24.1
	Muslim	213	37.4
	Protestant	203	35.7
	Other**	16	2.8
Family size	<5	432	75.9
	> = 5	137	24.1
Age of the child in month	24–35	250	43.9
	36–47	204	35.9
	48–59	115	20.2
Sex of the child	Male	291	51.1
	Female	278	48.9
ANC follow up	Yes	534	93.8
	No	35	6.2
Place of deliver	Health facility	349	61.3
	At Home	220	38.7
Vaccination status	Incomplete	153	26.9
	Complete	416	73.1
Had diarrhea in the last 2 weeks	Yes	166	29.2
	No	403	70.8
Had fever in the last 2 weeks	Yes	195	34.3
	No	374	65.7
Had Cough/ARI in the last 2 weeks	Yes	172	30.2
	No	397	69.8
Child DDS	<4 food Groups	305	53.6
	> = 4Food groups	264	46.4
Household Wealth status	Lowest	197	34.6
	Second	38	6.7
	Meddle	138	24.3
	Fourth	116	20.4
	highest	80	14.1
Source of drinking water	Improved water	301	52.9
	Non improved water	268	47.1
Sanitation	Improved	383	67.3
	Non improved	186	32.7

* Others-Daily laborers, students

* * Others-Wake feta and Catholic, ANC-Ant Natal Care.

ARI-Acute Respiratory Infection, DDS-Dietary Diversity Score.

Two hundred fifty (43.9%) of the children were between the ages of 24 and 35 months. Of the children involved in the study, more than one-half, 318 (55.9%) were male children. Five hundred thirty-four (93.8%) of the mothers had attended ANC at least one or more. Nearly 349 (61.3%) of the mothers gave birth at a health facility. Almost three-fourths (73.1%) of the children completed their vaccination. Of the total children, 166 (29.2%), 195 (34.3%) and 172 (30.2%) of them had diarrhea, fever and ARI/cough in the last 2 weeks respectively. More than 50% of the children had dietary diversity scores of less than four food groups. One hundred ninety seven (34.6%) and 138 (24.3%) of the total respondents were in the lowest and middle wealth indexes, respectively. In terms of drinking water and sanitation, 268 (47.1%) of respondents used non-improved sources of water, while 186 (32.7%) used non-improved sanitation **(Table 2).**

### CIAF and traditional indices were used to assess under-nutrition in preschool-aged children

According to CIAF criteria, 50.8% of pre-school aged children were undernourished. Based on conventional anthropometric indices such as wasting, stunting and underweight, the prevalence of under-nutrition was found to be lower. 12.8% were wasting, 42.4% were stunting and 27.9% were underweight. Based on sex-specific cases of stunting, 46.4% were observed among female preschool children. The prevalence of age-specific wasting, stunting, underweight and CIAF were observed among 48–59 months (14.8%), 24–35 months (42.8%), 48–59 months (33%) and 24–35 months (52.4%) respectively **(Table 3).**

**Table 3:** The prevalence of growth failure by sex and age group among preschool children in rural area of Ilu Aba Bor Zone, Southwest Ethiopia, 2019.

Variables		Wasting	Stunting	Underweight	CIAF
		N (%)	N (%)	N (%)	N (%)
**Sex**	Male	34(11.7)	112(38.5)	71(24.4)	134(46.0)
	female	39(14.0)	129(46.4)	88(31.7)	155(55.8)
**Age of the child in month**	24–35	33(13.2)	107(42.8)	73(29.2)	131(52.4)
	36–47	23(11.3)	85(41.7)	48(23.5)	98(48.0)
	48–59	17(14.80	49(42.6)	38(33.0)	60(52.2)
**Total**		73(12.8)	241(42.4)	159(27.9)	289(50.8)

### Preschool children’s anthropometric failure composite index

Among the subgroups of CIAF (B-Y) with under-nutrition, group F (stunting only) was the highest (22.5%), and children in group B (wasting only) had the lowest prevalence (0.4%) of under-nutrition. The prevalence of under-nutrition among groups C (wasting and underweight), D (wasting, stunting and underweight), E (Stunting and underweight) and Y (underweight only) was 3.7%, 8.3%, 11.1% and 4.9% respectively. Of the children, 48.7% had anthropometric failure group A (no failure) (**Table 4**).

**Table 4:** Anthropometric failure composite index among preschool children in rural Ilu Aba Bor Zone, Southwest Ethiopia, 2019.

Group	Description of the failure	Age in month			Sex		Total
		24–35	36–47	48–59	Male	Female	
**A**	No failure	119(47.6)	103(50.5)	55(47.8)	154(48.4)	123(49.0)	277(48.7)
**B**	Wasting only	0	2(1.0)	0	0	2(0.8)	2(0.4)
**C**	Wasting and underweight	11(4.4)	4(2.0)	6(5.2)	13(4.1)	8(3.2)	21(3.7)
**D**	Wasting, stunting and underweight	22(8.8)	14(6.9)	11(9.6)	24(7.5)	23(9.2)	47(8.3)
**E**	Stunting and Underweight	27(10.8)	20(9.8)	16(13.9)	32(10.1)	31(12.40	63(11.1)
**F**	Stunting only	58(23.2)	48(23.5)	22(19.1)	77(24.2)	51(20.3)	128(22.5)
**Y**	Underweight only	13(5.2)	10(4.9)	5(4.3)	15(4.7)	13(5.2)	28(4.90
**CIAF**		131(52.4)	98(48.0)	60(52.2)	161(50.6)	128(51.0)	189(50.8)

### Factors associated with the composite index of anthropometric failure

The composite index of anthropometric failure was significantly associated with the child’s sex, family size, caregiver’s occupational status, acute respiratory infection, source of drinking water, and dietary diversity score in a multivariable logistic regression study **(Table 5).**

**Table 5:** Factors associated with overall under-nutrition of preschool aged children in rural areas Ilu Abba Bor, South west Ethiopia, 2019.

Variable		CIAF		COR(95% CI)	AOR(95% CI)	P-value
		Failure	No failure			
Sex of the Child	Male	134	157	1	1	
	female	155	123	1.476(1.061,2.054)	1.51(1.076,2.12)	0.017
Educational status of caregiver	Can’t read and write	49	42	1.697(.71,4.056)	1.133(0.442,2.903)	0.794
	Can read and write	28	22	1.851(.716.4.783)	1.632(0.611,4.361)	0.329
	Grade 1–4	48	43	1.624(.68,3.88)	1.494(0.608,3.669)	0.382
	Grade 5–8	84	83	1.472(.645,3.361)	1.385(0.584,3.286)	0.459
	Grade 9–12	69	74	1.356(.589,3.125)	1.283(0.54,3.053)	0.573
	Tertiary education	11	16	1	1	
Marital status of the caregivers	Single	20	20	1	1	
	Married	239	245	0.976(0.512,1.859)	0.951(0.49,1.847)	0.882
	Widowed	12	5	2.4(0.713,8.077)	1.694(0.484,5.927)	0.409
	Divorced	18	10	1.8(0.668,4.848)	1.48(0.535,4.092)	0.45
Vaccination status of the child	Incomplete	84	69	1.253(0.863,1.818)	1.266(0.862,1.86)	0.228
	Complete	205	211	1	1	
Family size	<5	205	227	1	1	
	> = 5	84	53	1.755(1.186,2.598)	1.78(1.19,2.663)	0.005
ARI in the last 2 weeks	Yes	103	69	1.693(1.178,2.435)	1.767(1.216,2.566)	0.003
	No	186	211	1	1	
Source of drinking water	Improved	140	161	1	1	
	Non improved	149	119	1.44(1.034,2.004)	1.484(1.056,2.085)	0.023
DDS	<4group	169	136	1.491(1.071,2.077)	1.5(1.066,2.112)	0.02
	> = 4group	120	144	1	1	

ARI-Acute Respiratory Infection, DDS-Dietary Diversity Score, COR-Crude Odds Ratio, AOR-Adjusted Odds ratio, CIAF- Composite Index of Anthropometric Failures.

Female preschool children had a significantly higher risk of composite index of anthropometric failure (AOR = 1.51, CI: 1.076,2.12); preschool aged children living in families of five or more (AOR = 1.78, CI: 1.19,2.663); children who had an acute respiratory infection in the previous two weeks (AOR = 1.767, CI: 1.216,2.566); children whose households used an unimproved source of drinking water (AOR = 1.484, CI: 1.056,2.085) and preschoolers with a dietary diversity score of four or more food groups (AOR = 1.5, CI: 1.066,2.112) **(Table 5).**

## Discussion

Since conventional indices underestimate the prevalence of under-nutrition and do not provide the overall prevalence of under-nutrition in children, this finding provides new estimates of under-nutrition prevalence in preschool aged children using aggregate measures of anthropometric failure. This can be avoided by using the composite index of anthropometric failure as an aggregate indicator [28]. The prevalence of under-nutrition was high in the current study, suggesting that the CIAF is a public health problem in the study area. The variables that were significantly associated with under-nutrition were the child’s sex, family size, caregiver’s occupational status, acute respiratory infection, source of drinking water, and dietary diversity score.

Using a composite index of anthropometric failure, the current study found that 50.8% of preschool-aged children were undernourished. This result is in line with studies conducted in Malawi and Purba Medinipur, West Bengal, India, which found a 50.6% and 50.2 percent prevalence of CIAF, respectively [28, 29]. The current result is also comparable to studies conducted in Ethiopia (49%) [30] and various parts of India; Assam (51%) [31] and Angenwadi (51.8%) [32]. However, the current findings were higher than those found in Ethiopia (45.96%) [33], Tanzania (38.2%) [34], and various parts of India, including Parwano and Himacha Pradesh (31.9%) [35], rural areas of west Bengal (32.7%) [36], Kolkata, west Bengal (41.2%) [37] and Sonowal Kachari (48.6%) [38]. When compared with the prevalence of under-nutrition among Argentineans, (15.1%) [39], In the current research, the prevalence of malnutrition was much higher. Differences in socioeconomic, cultural, and child feeding patterns, seasonal variance in study time, and age group of study population and study setting could all contribute to the disparity.

The prevalence of under-nutrition found in this study was lower than that found in other areas of India, including the Parganas district, Jamma, Delhi’s resettlement colony, Angra City, Northern Odisha, Aligani’s Anganwadi Centers, and Chhattigarh, where the prevalence of under-nutrition was (61.3%), (73.2%), (62%), (60.04%), (54.4%), and (57%) [40–46]. It’s also lower than the prevalence reported from Yemen, where under-nutrition was found to be prevalent (70.1%) [47]. The age of the study subjects included in the study could be a possible explanation for the discrepancy. Children aged 2–5 years were included in this study, while children aged less than 2 years were included in previous studies. Female children were found to have a higher risk of malnutrition than male children, according to this report. The findings were consistent with a study conducted in Ethiopia, which found that female children were malnourished more than male children [48]. However, the present finding was inconsistent with the previous studies [48, 49] in which male children were more likely to be under-nourished than female children. The distinction may be due to the fact that childhood morbidity is higher in male children than in female children [49].

When compared to children with less than five family members, children with five or more family members were more likely to be undernourished. This result was in line with a study from Ethiopia’s Afar region, which found that the size of a household’s family was closely associated with wasting [50]. The current study found that preschool-aged children who had an acute respiratory infection in the previous two weeks were more likely to be undernourished than children who had not had an acute respiratory infection. This was supported by a study conducted in Burkina Faso and the urban slums of Agra City, which found that the ARI was significantly linked to child malnutrition [43, 51].

According to the findings, the source of drinking water was a major factor in under-nutrition, with children from families with unimproved water sources being more likely to be undernourished than children from families with improved water sources. The findings were in line with those of an Ethiopian analysis [30, 51, 52]. The researchers also found that preschool-aged children with DDS < 4 food groups were more likely to be undernourished than children with DDS > = 4 food groups. The current findings differ from those of previous research [42, 48]. The socioeconomic characteristics of the study population and study period may be a factor. Despite the fact that a systematic effort was made to determine the overall prevalence of under-nutrition among pre-school aged children using CIAF, the research has some limitations.

Although a pretest, instrument calibration, and close supervision were used to reduce bias, there were still measurement errors in gathering anthropometric data in this research. Furthermore, recall bias can occur when reporting a child’s dietary intake in the previous 24 hours as well as reporting child morbidity in the previous two weeks. To reduce recall bias, data collectors were trained how to ask caretakers/mothers about their child’s feeding patterns. Furthermore, due to the cross-sectional nature of the study design, determining the possible temporal relationship was difficult. While various factors causing under-nutrition in preschool-aged children were examined, details on some possible confounders such as the mother’s nutritional status, age at first pregnancy, birth weight and height of the infant, and some paternal characteristics were not examined.

## Conclusions

Our findings showed that pre-school aged children in the study area have a high prevalence of CIAF. The child’s sex, family size, ARI within the last two weeks, and dietary diversity score were all found to be significantly associated with CIAF. Children’s nutritional status in rural areas should be strengthened by adequate nutrition intervention programs, according to experts. To encourage the use of family planning, the prevention of infectious diseases, and the diversification of dietary choices, health education is required. Additional research should be done to look at related factors that were not included in this report. We also suggest that the government adapt CIAF as a metric for assessing children’s nutritional status in order to estimate the overall prevalence of malnutrition.

## Supporting information

S1 File Questionnaires. (DOCX)

S2 File Data. (SAV)

## Abbreviation

WHO: World Health Organization
CIAF: Composite Index of Anthropometric failure
AOR: Adjusted Odds Ratio
COR: Crude Odds Ratio
DDS: Dietary Diversity Score
ARI: Acute Respiratory Infection
EDHS: Ethiopia Demographic Health Survey
UNICEF: United Nations Children’s Fund
FAO: Food and Agricultural Organization
ANC: Antenatal Care
MDD: Minimum Dietary Diversity
FANTA: Food and Nutritional Technical Assistance
TEM: Technical error of Measurement
HAZ: Height for Age
WHZ: Weight for Height
WAZ: Weight for Age
SD: Standard Deviation
SPSS: Statistical Package for Social Science
VIF: Variance Inflation Factors
