# Supplementary material for: Anthropometric failures and its associated factors among preschool-aged children in a rural community in southwest Ethiopia
Source: PLoS One. 2021 Nov 29;16(11):e0260368. doi: 10.1371/journal.pone.0260368 (PMC8629177; doi:10.1371/journal.pone.0260368)
Supplement: S1 File — (DOCX) [file pone.0260368.s001.docx]

**Table 1 (A).** The questionnaire used for the survey on recovered COVID-19 patients

| **General information** | | | | |
| --- | --- | --- | --- | --- |
| Age (in years) |  | | | |
| Nationality |  | | | |
| Gender | Male | | Female | |
| When infected by COVID-19 (Coronavirus) your diagnosis was by | Swab only without symptoms | Symptoms | Swab and symptoms | |
| **After you have been cured of COVID-19 Do you manifested any of the following symptoms (you can choose one or more symptom(s):** | | | | |
| I-General Symptoms | Degree | | | Number of days this symptom lasted with you |
| 1-Fatigue | Mild +1 | Moderate +2 | Severe +3 |  |
| 2- General Weakness | Mild +1 | Moderate +2 | Severe +3 |  |
| II- Skin and Musculoskeletal symptoms | Degree | | | Number of days this symptom lasted with you |
| 1-Muscle aches | Mild +1 | Moderate +2 | Severe +3 |  |
| 2-Joint pains | Mild +1 | Moderate +2 | Severe +3 |  |
| 3-Skin Rash | Mild +1 | Moderate +2 | Severe +3 |  |
| III-Psychological and Neurological symptoms | Degree | | | Number of days this symptom lasted with you |
| 1-Headache | Mild +1 | Moderate +2 | Severe +3 |  |
| 2-Mood changes | Mild +1 | Moderate +2 | Severe +3 |  |
| 3-Insomnia | Mild +1 | Moderate +2 | Severe +3 |  |
| 4-Paraesthesia and anesthesia | Mild +1 | Moderate +2 | Severe +3 |  |
| IV-Special sense symptoms | Degree | | | Number of days this symptom lasted with you |
| 1-Hearing problems | Mild +1 | Moderate +2 | Severe +3 |  |
| 2-Visual disturbances | Mild +1 | Moderate +2 | Severe +3 |  |
| 3-Dry eyes | Mild +1 | Moderate +2 | Severe +3 |  |
| 4-Loss of smell | Mild +1 | Moderate +2 | Severe +3 |  |
| 5-Loss of taste | Mild +1 | Moderate +2 | Severe +3 |  |
| V-Respiratory system symptoms | Degree | | | Number of days this symptom lasted with you |
| 1-Cough | Mild +1 | Moderate +2 | Severe +3 |  |
| 2-Breathlessness and chest tightness | Mild +1 | Moderate +2 | Severe +3 |  |
| VI-Gastrointestinal symptoms | Degree | | | Number of days this symptom lasted with you |
| 1-Lack of appetite | Mild +1 | Moderate +2 | Severe +3 |  |
| 2-Nausea | Mild +1 | Moderate +2 | Severe +3 |  |
| 3-Diarrhea | Mild +1 | Moderate +2 | Severe +3 |  |
| 4-Abdominal pain | Mild +1 | Moderate +2 | Severe +3 |  |

**Table 1(B).** An Arabic version of the questionnaire used for the survey on recovered COVID-19 patients

| **معلومات عامة:** | | | | |
| --- | --- | --- | --- | --- |
|  | العمر (بالسنوات) | | | |
|  | الجنسية | | | |
| أنثى | ذكر | | الجنس | |
| بالمسحة والأعراض معا | بالأعراض فقط | بالمسحة فقط دون ظهور اعراض | كيف تم تشخيصك عند اصابتك بالكورونا | |
| **بعد شفائك من الكورونا هل عانيت أحد من هذه الأعراض او أكثر (**يمكنك اختيار أكثر من عرض إذا كان ينطبق عليك) | | | | |
| مدة استمرار هذا العرض بالأيام | درجة الإحساس بهذا العرض | | | **أعراض عامة** |
|  | بدرجة شديدة+3 | بدرجة متوسطة+2 | بدرجة بسيطة+1 | 1-الإحساس بالإعياء: |
|  | بدرجة شديدة+3 | بدرجة متوسطة+2 | بدرجة بسيطة+1 | 2- الإحساس بالضعف العام: |
| مدة استمرار هذا العرض بالأيام | درجة الإحساس بهذا العرض | | | **اعراض خاصة بالعضلات والمفاصل والجلد** |
|  | بدرجة شديدة+3 | بدرجة متوسطة+2 | بدرجة بسيطة+1 | 1- الإحساس بالآم بالعضلات: |
|  | بدرجة شديدة+3 | بدرجة متوسطة+2 | بدرجة بسيطة+1 | 2- الإحساس بالآم المفاصل: |
|  | بدرجة شديدة+3 | بدرجة متوسطة+2 | بدرجة بسيطة+1 | 3-ظهور طفح جلدي |
| مدة استمرار هذا العرض بالأيام | درجة الإحساس بهذا العرض | | | **اعراض نفسية-عصبية** |
|  | بدرجة شديدة+3 | بدرجة متوسطة+2 | بدرجة بسيطة+1 | 1- الإحساس بالصداع: |
|  | بدرجة شديدة+3 | بدرجة متوسطة+2 | بدرجة بسيطة+1 | 2- تغيرات المزاج: |
|  | بدرجة شديدة+3 | بدرجة متوسطة+2 | بدرجة بسيطة+1 | 3-الشعور بالأرق: |
|  | بدرجة شديدة+3 | بدرجة متوسطة+2 | بدرجة بسيطة+1 | 4-الإحساس بالتنمل او التخدير |
| مدة استمرار هذا العرض بالأيام | درجة الإحساس بهذا العرض | | | **اعراض خاصة بالحواس** |
|  | بدرجة شديدة+3 | بدرجة متوسطة+2 | بدرجة بسيطة+1 | 1- مشاكل في السمع: |
|  | بدرجة شديدة+3 | بدرجة متوسطة+2 | بدرجة بسيطة+1 | 2- اضطرابات الرؤية: |
|  | بدرجة شديدة+3 | بدرجة متوسطة+2 | بدرجة بسيطة+1 | 3-الشعور بجفاف العين: |
|  | بدرجة شديدة+3 | بدرجة متوسطة+2 | بدرجة بسيطة+1 | 4-فقدان حاسة الشم: |
|  | بدرجة شديدة+3 | بدرجة متوسطة+2 | بدرجة بسيطة+1 | 5-فقدان التذوق |
| مدة استمرار هذا العرض بالأيام | درجة الإحساس بهذا العرض | | | **اعراض خاصة بالجهاز التنفسي** |
|  | بدرجة شديدة+3 | بدرجة متوسطة+2 | بدرجة بسيطة+1 | 1- سعال: |
|  | بدرجة شديدة+3 | بدرجة متوسطة+2 | بدرجة بسيطة+1 | 2- الشعور بضيق التنفس وضيق الصدر: |
| مدة استمرار هذا العرض بالأيام | درجة الإحساس بهذا العرض | | | **اعراض خاصة بالجهاز الهضمي** |
|  | بدرجة شديدة+3 | بدرجة متوسطة+2 | بدرجة بسيطة+1 | 1- قلة الشهية: |
|  | بدرجة شديدة+3 | بدرجة متوسطة+2 | بدرجة بسيطة+1 | 2 الشعور بالغثيان: |
|  | بدرجة شديدة+3 | بدرجة متوسطة+2 | بدرجة بسيطة+1 | 3-إسهال: |
|  | بدرجة شديدة+3 | بدرجة متوسطة+2 | بدرجة بسيطة+1 | 4-الم بالبطن |
